# Genotype-phenotype correlations in FSHD

**DOI:** 10.1186/s12920-019-0488-5

**Published:** 2019-03-13

**Authors:** Nikolay Zernov, Mikhail Skoblov

**Affiliations:** 1 Research Center for Medical Genetics, Moscow, Russia; 2 School of Biomedicine, Far Eastern Federal University, Vladivostok, Russia

**Keywords:** Inherited disease, FSHD, D4Z4, Genotype-phenotype correlation, Methylation, Anticipation

## Abstract

**Background:**

Facial-scapular-humeral myodystrophy Landouzy-Dejerine (FSHD) is an autosomal dominant disease, the basis of its pathogenesis is ectopic expression of the transcription factor DUX4 in skeletal muscle. There are two types of the disease: FSHD1 (MIM:158900) and FSHD2 (MIM: 158901), which have different genetic causes but are phenotypically indistinguishable. In FSHD1, partial deletion of the D4Z4 repeats on the 4th chromosome affects the expression of DUX4, whereas FSHD2 is caused by the mutations in the protein regulating the methylation status of chromatin - SMCHD1. High variability of clinical picture, both intra - and inter-family indicates a large number of factors influencing clinical picture. There are key genetic, epigenetic and gender factors that influence the expressivity and penetrance of the disease. Using only one of these factors allows just a rough prediction of the course of the disease, which indicates the combined effect of all of the factors on the DUX4 expression and on the clinical picture.

**Results:**

In this paper, we analyzed the impact of genetic, epigenetic and gender differences on phenotype and the possibility of using them for disease prognosis and family counselling.

**Conclusions:**

Key pathogenesis factors have been identified for FSHD. However, the pronounced intra - and inter-family polymorphism of manifestations indicates a large number of modifiers of the pathological process, many of which remain unknown.

## Introduction

Facioscapulohumeral muscular dystrophy (FSHD) is a disease from a group of hereditary progressive skeletal muscle dystrophies. In most cases, FSHD is characterized by progression of dystrophic changes in the cranio-caudal direction with asymmetric lesions of facial muscles, shoulder girdle, shoulders and legs. The prevalence of the disease in different populations varies from 1:20′000 to 1:14′000, conceding only to Duchenne muscular dystrophy and myotonic dystrophy [1–4].

The linkage between FSHD and subtelomeric region of chromosome 4q35 has been shown for the first time in the works of the 1990s [5–8]. In 4q35 region, an array of macrosatellite repeats was found, each named D4Z4 and consisting of 3.3 thousand base pairs [9]. The D4Z4 sequence is GC-rich (~ 70%) and is normally hypermethylated [10–13]. D4Z4-like repeats are widely distributed in the genome, the maximum homology is between sequences of the D4Z4 repeats on the chromosomes 4 and 10 [14]. This fact is explained by the translocation of the D4Z4 repeat area from 4-th to 10-th chromosome in the course of hominid evolution [15]. Despite this, FSHD is only linked to the D4Z4 repeats on the 4th chromosome.

Deeper understanding of this linkage came after further investigation of the 4th chromosome D4Z4 repeats sequence. It was shown that each D4Z4 repeat on the 4th chromosome contains several coding sequences, of which the protein-coding retrogene *DUX4* was described to have the most prominent role in the development of FSHD [16–18]. Snider et al. [18] using RT-PCR observed two isoforms of DUX4 mRNA in human tissues: DUX4fl and DUX4s. DUX4fl is abundantly expressed in testis, but is absent in any other examined adult tissues. Whereas DUX4s was observed in ovary, heart, skeletal muscles and liver. DUX4 protein is a putative transcription factor containing two homeodomains [16, 17]. Its role was shown for different processes: activation of apoptotic pathways such as p-53, caspase-3, and caspase-7 mediated [19]; inhibition of differentiation and myogenesis in C2C12 cells [20]; inducing set of genes expressed in germ cells [21] and PITX1 induced cytotoxicity [22]. However, the precise pathway by which DUX4 induces myodistrophic changes in FSHD is still controversial.

FSHD linked contracted 4q35 D4Z4 array shows pronounced hypomethylation and chromatin relaxation [23]. Interestingly, such epigenetic status of contracted 4q35 D4Z4 array is not enough for development of FSHD. FSHD-linked allele is always in *cis* with the so-called 4qA haplotype – the sequence distal to the D4Z4 array. Population studies have found two predominant haplotypes distal to the D4Z4 array of the region 4q35, designated as 4qA and 4qB. The frequency of their occurrence is approximately 50% [15, 24]. An important difference between these haplotypes is that only 4qA variant contains the sequence of polyadenylation signal for DUX4 mRNA and is associated with the expression of the DUX4 protein in skeletal muscles of adults [21].

To date there are two types of the FSHD: FSHD1 (MIM: 158900) and FSHD2 (MIM: 158901). FSHD1 is caused by the partial deletion of D4Z4 array in *cis* with the 4qA haplotype. Thus the number of residual D4Z4 repeats varies in the range from 1 to 10. FSHD1 is characterized by autosomal dominant inheritance and accounts for > 95% of all FSHD cases [3, 9, 25, 26]. FSHD2 occurs in less than 5% of cases [27]. Of these, approximately 80% of cases are caused by mutations in the SMCHD1 gene - the regulator of the chromatin methylation status; in particular the array of D4Z4 repeats [28]. FSHD2 has digenic mode of inheritance, in view of the fact that mutations in the SMCHD1 gene on chromosome 18 are inherited regardless of contraction of the D4Z4 repeat in *cis* with 4qA haplotype [26].

Although genetic causes are different, both types of FSHD show relaxation of chromatin of the D4Z4 array on the 4qA haplotype and activation of the ectopic expression of *DUX4* retrogene from the last D4Z4 repeat. As a result, the phenotypic signs for both types of FSHD are identical [29].

The pronounced variability of FSHD clinical picture makes it difficult to predict the course of the disease and to consult families. However, current findings show that there is a correlation between the clinical picture and such factors as follows: number of D4Z4 repeats on contracted 4qA haplotype; level of methylation of the D4Z4 array; gender and age of onset. Therefore, today, it is possible to give the most accurate prediction considering all the above-mentioned factors.

### Scoring of clinical severity

To date, there are no specific, fully validated scores for assessing the severity of the FSHD. The most frequently used methods of assessment are as follows: manual tests; quantitative tests; age of onset, combined methods, considering the degree of muscle weakness, gender, age and height. Lamperti et al. [30] suggested using a scale that takes into account both the degree of muscle damage and the progression of the pathological process. In addition, the authors assessed the reproducibility of the results by different neurologists in a sample of 69 patients and showed high concordance of the results (kappa value 0.774). However, their work has not been widely used in subsequent studies.

Since FSHD is a slowly progressing myopathy, it is difficult for the patient to determine the exact age of onset. Age of onset is usually determined retrospectively and often depends on the memories and perceptions of the patient and their family. Therefore, van Overveld et al. [12] has upgraded the scale published by Ricci et al. [31] to correct the calculations of severity depending on the age at the time of examination. This scale is the most frequently used and includes 10 possible values (from 0.5 to 5). The minimum assessment is given in the presence of weakness only of facial muscles, the maximum-in case of pelvic muscles involvement. Furthermore, the calculations include the age of the patient at the time of the examinations and display the final values representing the severity of the disease. However, it is worth noting that this scale may not be suitable for assessing the severity in case of the dystrophic process progressing in the non-classical direction.

Several authors draw attention to the correlation of the onset age with the rate of progression of the pathological process and distinguish three clinical variants of the course: early onset up to 10 years with rapid progression and early disability [32–34]; classical debut in the second decade of life with typical progression [35, 36]; late variant with the onset after 40 years and slow progression with minimal clinical signs [35, 36].

To our knowledge, the studies of phenotype-genotype correlations were performed mostly for FSHD1. FSHD1 is characterized by a pronounced intra-and inter-family polymorphism of clinical picture, as well as a high frequency of sporadic cases with severe manifestations [36]. In view of this, in many studies the authors have investigated samples of the two types: familial and sporadic cases.

### Influence of the number of D4Z4 repeats on the severity of FSHD

In most studies of the influence of genetic factors on phenotype, the inverse correlation between the number of the D4Z4 repeats on the 4th chromosome and the severity of clinical manifestations was established. Ricci et al. [31] have shown that the most pronounced correlation was for the patients with the number of repeats in the range from 1 to 2. Severe muscle damage of lower extremities was observed for 100% patients with the number of repeats from 1 to 2, 53% with the number of repeats from 3 to 4 and 19% in the group of patients with a repeat count of 5 or more. Interestingly, in 13 sporadic cases, the severity was statistically higher than in 132 family cases.

Lunt et al. [37] cases have shown that the length of the D4Z4 array is in direct correlation with the age of the onset of the disease in 14 family and 25 isolated FSHD. The smallest number of the D4Z4 repeats and an early onset are found more often in the group of isolated cases (r = 0.56; *P* < 0.001), a weak correlation was observed in the group with the “border line” number of the D4Z4 repeats in the range of 8–10 units [15, 38, 39]. The same observation has been made by Butz et al. [40] on patients with the “border line” number of the D4Z4 repeats from 8 to 10. No correlation between the severity of the disease and the number of repeats has been found for 12 family and 27 sporadic cases, and clinical manifestations ranged from severe to asymptomatic carrier.

About one fifth of patients with FSHD1 become dependent on the wheelchair during the disease progression, among them the most common are individuals with the number of the D4Z4 repeats from 1 to 3 [31, 35, 37, 40, 41]. Cases of asymptomatic carriage or minimal signs also represent one-fifth of all cases of FSHD1 and are characteristic for these individuals 8 to 10 D4Z4 repeats.

In our opinion, these observations are consistent with the presence of three clinical variants of FSHD. Thus, cases with early onset, rapid progression and disability are most often found in the group of sporadic cases with the number of D4Z4 repeats from 1 to 3, while patients with the number of repeats from 8 to 10 are characterized by late onset, slow progression and minimal clinical manifestations.

Interestingly, the frequency of pathogenic contraction of the D4Z4 array on the 4qA haplotype is approximately 1–2% [29, 39] in the population of healthy individuals, which can be explained by incomplete penetrance, insufficient clinical examination, delayed onset of the disease or the influence of epigenetic factors. Somatic mosaicism is observed in about 10–30% of all FSHD1 cases, and in de novo cases the number of patients with somatic mosaicism reaches 50% [42]. It is possible that the mosaicism (of D4Z4 alleles) is also a common phenomenon in populations of healthy individuals.

### The methylation level of the D4Z4 repeats array and the severity of the disease

The pronounced polymorphism of FSHD clinical picture and the high percentage of GC content in sequence of D4Z4 repeats indicate a significant role of CpG methylation level to regulation of DUX4 expression and, consequently, the severity of clinical signs. The first attempts to explore the correlation between the methylation level and the number of the D4Z4 repeats was performed by Tsien et al. [43] on cell cultures, semen, and muscle biopsies of patients with FSHD using restriction enzymes SmaI, MluI, SacII, and EagI. The authors did not observe any significant difference in the methylation of D4Z4 repeats by comparing healthy individuals and patients with FSHD, as well as within the group of the patients with different number of D4Z4 repeats.

However, in a later study van Overveld et al. [13] using the methyl-sensitive restriction enzymes, such as BsaAI and FseI, have observed a difference in the methylation of D4Z4 repeats between healthy individuals and patients. In addition, the methylation levels among patients with different number of repeats were observed [10, 12]. In a subsequent study, these observations were confirmed on blood mononuclear samples using restriction by FseI, and a direct dependence of the methylation levels on the number of D4Z4 repeats was also established [38]. Moreover, the methylation levels of the D4Z4 repeats are correlated with such marker of the chromatin compactization as histone H3 methylation. For both types of FSHD, the inverse dependence of the severity of clinical manifestations on the levels of methylation of the D4Z4 repeats on 4qA chromosome was shown [38].

These correlations are most pronounced in groups of patients with the number of the D4Z4 repeats from 1 to 3, while in patients with the number of the D4Z4 repeats from 8 to 10 the correlation is weak. It is worth noting that in the group of phenotypically healthy individuals with the number of the D4Z4 repeats from 8 to 10, the level of methylation of the D4Z4 array is higher than for patients with the same number of the D4Z4 repeats [38, 44, 45]. This may indicate the presence of additional genetic and epigenetic modifiers responsible for changing the status of the chromatin of the D4Z4 repeats array, and, as a consequence, penetrance and severity of FSHD. Perhaps this explains the high frequency of individuals with the number of the D4Z4 repeats from 8 to 10 in a healthy population.

In about 80% of FSHD2 cases, the level of methylation of D4Z4 repeats array depends not only on their number, but also on the mutations in the SMCHD1. SMCHD1 is a large protein (2005 amino acids in humans) consisting of three main domains: an N-terminal GHKL (gyrase, Hsp90, histidine kinase, MutL)- ATPase domain, C-terminal structural maintenance of chromosomes (SMC) domain and connecting central domain sharing no homology with other characterized proteins.

Blewitt et al. [46] have demonstrated that SMCHD1 has a role in the maintenance of X chromosome inactivation and the hypermethylation of CpG islands associated with the inactive X chromosome. In SMCHD1-knock down experiments on myotubes carrying permissive 4qA haplotype DUX4 mRNA increased levels and transcriptional activation of known DUX4 target genes as well as D4Z4-chromatin relaxation were observed [28, 47]. There is a broad spectrum of types of the mutations and localizations of the mutations in *SMCHD1* spanning whole gene. It is known that SMCHD1 subunits form homodimers [48], so mutations could affect not only the functioning of SMCHD1 but also the process of homodimerization. Therefore, it is believed that nonsense mutations lead to haplo-insufficiency of the protein and milder manifestations due to the presence of normal SMCHD1 homodimers. While missense mutations exert a dominant negative effect due to the presence of the mutant subunit in most SMCHD1 homodimers [38]. This can explain why nonsense mutation in the SMCHD1 has been found in patients with the number of the D4Z4 repeats from 11 to 16, while missense mutations with a dominant-negative effect might occur in patients with number of the D4Z4 repeats > 20.

Another interesting observation is coexisting FSHD1 and FSHD2 [47, 49]. Especially in these cases, the mutations in SMCHD1 play a role as modifiers of disease severity in FSHD1 patients comparing to their relatives without SMCHD1-mutations. This fact clearly demonstrates that SMCHD1 mutations influence the disease severity through the level of D4Z4 array methylation.

**Fig. 1:**
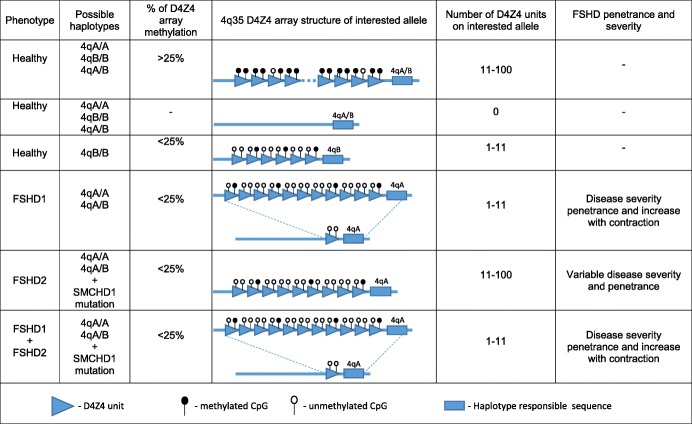
Variation of severity and penetrance of the disease, depending on genetic and epigenetic factors

Overview of genetic and epigenetic factors influencing disease severity and penetrance are presented in Fig. 1.

### Anticipation

The phenomenon of anticipation is well known in the so-called “trinucleotide repeat expansion disorders”, for example, myotonic dystrophy. In this case, mutations are dynamic and a gradual increase in the number of trinucleotide repeats in several generations are observed. For FSHD the number of D4Z4 repeats are stable in consequent generations. The first study showing a phenomenon like anticipation was the work of Lunt et al. [37]. The authors observed a difference in the disease onset in a series of 3 generations, reaching an average difference of 12.3 years for the patients with the same number of the D4Z4 repeats, in the group of 14 families with FSHD. A similar picture was observed for 11 families: among 2 generations, the difference in the average onset of the disease was 13.7 years [50]. Tawil et al. [51] have used the data from the quantitative estimations of muscles strength adjusted for the age of FSHD patients to assess the anticipation. In that work, the authors have found a significant (r = 0.92, *p* < 0.004) correlation between the disease severity and the size of the 4q35-associated deletion. Also, when relative disease severity of parent-offspring pairs was compared among 2 generations of 23 families, the offspring was found to be affected significantly more severely (*p* = 0.011). However, the authors of the works mentioned above rightly point out that the data of the age of the disease onset was obtained from the patients’ reports, as well as that there could be no severe cases due to early mortality in older generations. Thus larger-scale studies are needed to confirm or reject FSHD anticipation.

### Gender difference

Before the genetic cause of the disease was revealed, many authors observed the predominance of female patients in groups with mild manifestations of FSHD [35, 52, 53]. The first work that showed the predominance of women in the group of asymptomatic carriers of pathogenic haplotype was the study of Ricci et al. [31]. However, the small group size (7 asymptomatic carriers) did not make it possible to assess the correlation. The following works [41, 53, 54] have shown the predominance of women in the larger groups of asymptomatic carriers and patients with minimal manifestations. The investigation of the possible methylation level correlation on the disease severity by Balog et al. [55] revealed that men predominate in the group with severe course and hypomethylated D4Z4 repeats. However, according to the work of Lemmers et al. [38] on a larger sample (> 500 patients), the levels of methylation between the sexes did not differ statistically.

## Conclusion

To date, key pathogenesis factors have been identified for FSHD. However, the pronounced intra - and inter-family polymorphism of manifestations indicates a large number of modifiers of the pathological process, many of which remain unknown.

Here we analyzed the impact of genetic and epigenetic factors on FSHD clinical manifestations. Existing numerous studies allow to conclude that the penetrance and the severity of FSHD are in inverse correlation with the number of D4Z4 repeats on the 4qA haplotype and with the level of methylation. Moreover, the smaller the residual number of the D4Z4 repeats, the smaller the variability of the clinical picture, while the influence of the epigenetic regulators on the disease severity is increased for broader sized D4Z4 arrays (8–11 units). Groups of asymptomatic carriers and patients with minimal clinical manifestations are predominantly represented by females. Although there is no exact agreed criteria for clinical prognosis of FSHD to date, most researchers conclude that the most accurate prediction could be made with the following data:

- age of onset, gender, and the family examination data
- scoring of the clinical severity
- the length of D4Z4 repeats on the 4qA chromosome in FSHD1, additionally for FSHD2 – the presence and the type of mutations in the *SMCHD1* gene;
- haplotype and methylation status of the contracted array of D4Z4 repeats of 4th chromosome.

In our opinion, the phenomenon similar to anticipation for FSHD is still controversial and requires observations on large samples to confirm or reject it. Studies conducted to search for the correlations allowed us to determine some of the modifiers of the pathological process in FSHD, but further studies are needed for more accurate prediction of the clinical picture. In addition, they would help to better understand the mechanisms of DUX4 expression activation and thereby help in the search of therapeutic targets.

## Abbreviations

FSHD: FacioScapuloHumeral muscular Dystrophy
mRNA: Messenger RiboNucleic Acid
RT-PCR: Reverse Transcription Polymerase Chain Reaction
